# The anterolateral ligament of the knee: unwrapping the enigma. Anatomical study and comparison to previous reports

**DOI:** 10.1007/s10195-016-0392-0

**Published:** 2016-02-09

**Authors:** Jonathan D. Kosy, Ashish Soni, Ramakrishnan Venkatesh, Vipul I. Mandalia

**Affiliations:** 1 Princess Elizabeth Orthopaedic Centre, Royal Devon and Exeter Hospital, Barrack Road, Exeter, Devon EX2 5DW UK; 2 Leeds Teaching Hospitals, Leeds, UK

**Keywords:** Anterolateral ligament, Knee anatomy, Anterior cruciate ligament reconstruction, Knee stability

## Abstract

**Background:**

It has been suggested that the anterolateral ligament (ALL) of the knee may have importance in limiting rotational instability, and reconstruction may prevent a continued pivot-shift following anterior cruciate ligament surgery. However, the anatomy of this ligament has not been consistently reported in recent publications. We describe our experience of cadaveric dissection with reference to other published work.

**Materials and Methods:**

Eleven fresh-frozen cadaveric knees were dissected using a standard technique. The ALL tissue was identified with internal rotation of the tibia and varus stress. Measurements were made using a digital caliper and details of the origin and insertion were recorded.

**Results:**

The ALL was identified in ten of the 11 cadavers. The only specimen in which it was not identified was found to also have an anterior cruciate ligament deficiency. The mean dimensions were: length 40.1 (± 5.53) mm, width 4.63 (± 1.39) mm, thickness 0.87 (± 0.18) mm. The femoral origin was posterior and proximal to the lateral collateral ligament attachment in six knees, anterior and distal in three knees, and at the same site in one knee. The tibial insertion was a mean 17.7 (± 2.95) mm from Gerdy’s tubercle (GT) and 12.3 (± 3.55) mm from the fibula head (FH). This was 59.5 (± 5.44) % from GT to FH.

**Conclusions:**

This anatomical data adds to previous information about the ALL. Our results support the finding that the ALL is a capsular thickening with meniscal attachment. The findings will help to guide the further work required to define the indications for reconstruction and appropriate grafts.

## Introduction

Recently, there have been multiple publications on the subject of the anterolateral ligament (ALL) of the knee. It has been proposed that this structure plays a role in limiting anterolateral rotational instability and that reconstruction, when combined with intra-articular anterior cruciate ligament (ACL) reconstruction, may be beneficial [1–6].

ACL reconstruction is generally a successful procedure with long-term outcomes of improved function and reduced meniscal injuries compared to the unreconstructed knee [7]. However, the desire to control rotational instability (demonstrated clinically with the pivot-shift) brought about the development of double-bundle reconstructions and, now, increased interest in extra-articular reconstruction [5, 8, 9]. Indeed, in a study where the stabilising structures of the knee were sequentially sectioned, it appeared that the anterolateral structures (rather than the posterolateral bundle of the ACL) had the largest role in controlling rotational stability [4].

The results of recent work, focussed on the anatomy of the anterolateral structures of the knee, were announced as the discovery of a new ligament—the ALL [10–12]. However, the presence of this structure had, previously, been described by other authors [3, 13–15] and, historically, had been reported (as far back as 1879) in various guises –– “pearly band” attached to Segond fracture [15], mid-third (lateral) capsular ligament [16–18], anterior oblique band of lateral collateral ligament [19, 20]. More recently, this structure has been described through anatomical dissection [1, 3, 10, 13, 14, 22], histological analysis (demonstrating the existence of parallel collagen bundles and nerve fibres consistent with a ligamentous structure) [1, 3], radiological studies [23–26], and in association with the pathognomonic Segond fracture seen in association with ACL injuries [27].

However, a level of confusion still exists, with conflicting reports being published. Whilst the ALL has been found in all specimens in some studies [1, 3, 12] reports have been as low as 50 % [22]. Some studies have demonstrated the ALL as a capsular structure with an attachment between the lateral meniscus [1, 3, 10, 12] but others have claimed that it is extra-capsular with no such attachment [13]. Furthermore, the dimensions of the ALL are variably described, with lengths from 37 to 59 mm reported [3, 13]. Therefore, in trying to investigate the role of extra-articular reconstruction (to treat the deficiency of this structure), it seems important to characterise the ALL more clearly. It is hoped that the resultant procedure will have a better outcome than previous attempts at extra-articular reconstruction where residual instability and degeneration, within the lateral compartment, were found to be unacceptably high [28–32].

The purpose of this paper is to describe our experience of looking for the ALL with cadaveric dissection. We describe our findings, with comparison to the results of previous studies, and make suggestions about further work. Our goal was to identify the structure that appeared to be controlling anterolateral rotation, characterise its dimensions and attachments, and interpret them in the context of previous work.

## Materials and methods

**Fig. 1:**
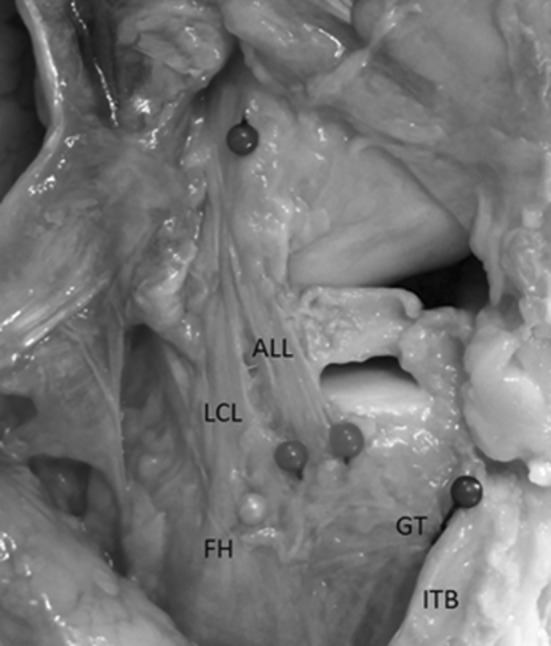
Photograph of dissected specimen. *ALL* anterolateral ligament, *LCL* lateral collateral ligament, *FH* fibula head, *GT* Gerdy’s tubercle, *ITB* iliotibial band

Eleven fresh-frozen cadaveric knees [nine female; two male; median age 79 years (range 71–88 years)] underwent a standardised anatomical dissection. The ALL was identified by using a dissection technique that closely mimicked that of Caterine et al. [1]. This is also similar to that used in previous studies [10, 13]. The lateral skin was removed as a large flap and the iliotibial band (ITB) was exposed from its insertion [Gerdy’s tubercle (GT)] to the mid-thigh. The ITB was transected 200 mm proximal to its insertion and care was taken to elevate this without damaging the deep structures. Loose connective tissue was removed to demarcate the anterolateral structures. The tibia was internally rotated, throughout the dissection, to identify structures under tension. The lateral collateral ligament (LCL) was defined (as an easily identifiable structure) and dissection proceeded anteriorly to isolate the tight structure (the ALL) and remove tissue not under tension in this internally rotated position. Once isolated, the attachments of the LCL and ALL were defined, along with the centre of the fibula head (FH) and GT (Fig. 1). Other groups have chosen to either remove the ITB from distal to proximal [3, 22] or the entire extensor apparatus [21]. We felt that both these techniques conferred increased risk of inadvertent damage to the ALL structure (due to the close proximity of structures around GT) so chose to elevate the transected ITB from proximal to distal.

Next, the dimensions of the ALL were recorded using a digital calliper (capacity = 150 mm, accuracy 0.01 mm). All measurements were made with the knee in 30 degrees of flexion and neutral rotation. The proximal attachment was defined in relation to the LCL. Internal rotation was then applied to the tibia to observe the effect on the identified tissue. Further dissection was then performed to demonstrate any attachments to the capsule and lateral meniscus. The presence of the ACL was then determined intra-articularly.

## Results

The results of each of the 11 dissections are displayed in Table 1.

**Table 1:** Results of dissection and measurement

Specimen	Age (years)	Sex	Side	Femoral origin (v. LCL)	Tibial insertion (mm from)		Length (mm)	Width (mm)	Thickness (mm)
					GT	FH			
1	84	F	R	AD	15.25	12.80	31.18	2.10	0.79
2	88	M	R	PP	20.60	8.54	40.51	2.35	0.84
3	79	F	R	AD	14.28	8.48	40.28	4.71	0.82
4	72	F	R	AD	16.67	10.85	48.02	5.38	1.05
5	72	F	R	PP	15.96	10.60	42.22	5.36	0.76
6	84	F	L	ALL not identified					
7	74	M	R	Same	17.74	17.52	39.70	6.09	0.69
8	85	F	R	PP	15.88	10.32	41.29	4.84	1.06
9	79	F	R	PP	16.04	10.10	41.78	4.91	0.68
10	71	F	L	PP	23.06	18.05	45.37	4.43	1.22
11	72	F	L	PP	21.35	15.48	30.28	6.14	0.76

*AD* anterodistal, *PP* posteroproximal, *LCL* lateral collateral ligament, *GT* Gerdy’s tubercle, *FH* fibula head

**Fig. 2:**
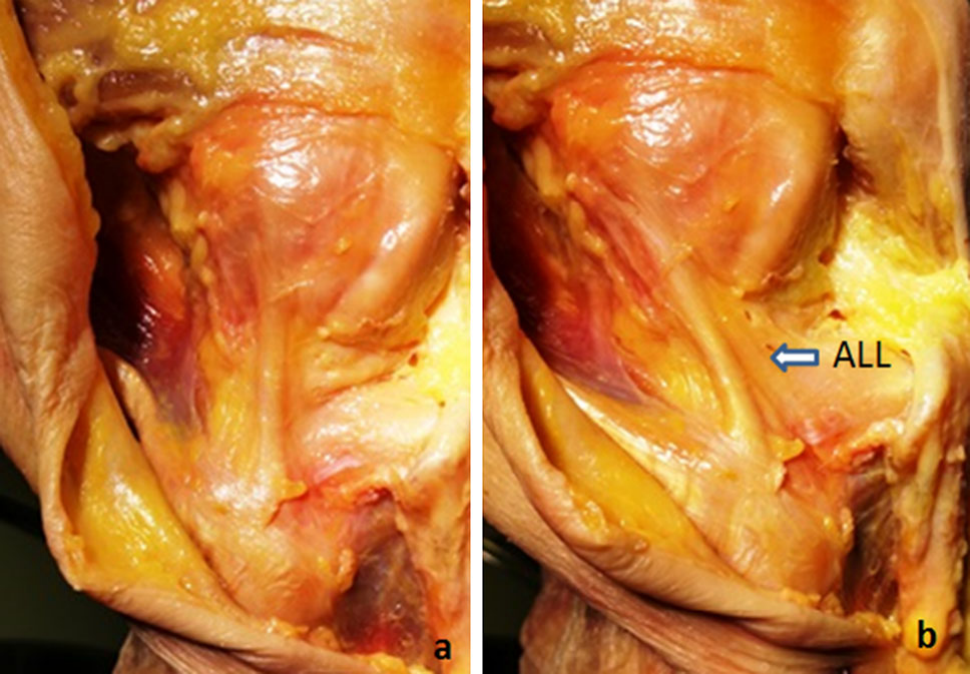
Photographs demonstrating the tightening of the ALL between **a** a neutral position and **b** with internal rotation of the tibia

We were able to identify the ALL in 10 of the 11 specimens (90.9 %). Of note, the specimen without an ALL was also found to be the only specimen without an intact ACL. On internal rotation of the tibia, in each case, there was both a palpable band of tissue that became taut and (on further dissection) the appearance of organised bundles running obliquely (Fig. 2).

The mean dimensions were: length 40.1 (±5.53) mm, width 4.63 (±1.39) mm, thickness 0.87 (±0.18) mm. The femoral origin was posterior and proximal to the lateral collateral ligament attachment in six knees, anterior and distal in three knees, and at the same site in one knee. The tibial attachment was found to be a mean 17.7 (±2.95) mm from the GT and 12.3 (±3.55) mm from the FH. This was 59.5 (±5.44) % from GT to FH.

**Fig. 3:**
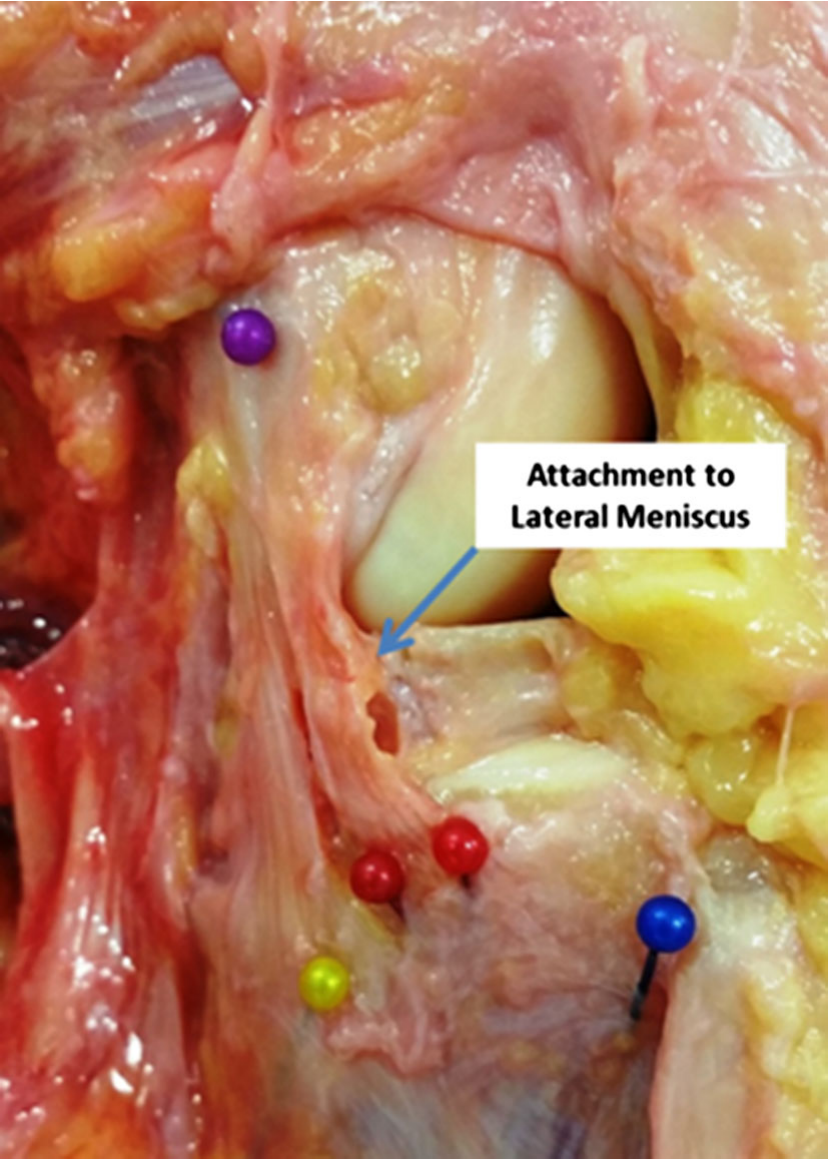
Photograph demonstrating attachment of the dissected ALL to the lateral meniscus

We found that it was difficult to decisively determine the borders of the ALL tissue, as it was continuous with the capsule, when identified, in all cases. We used the extent of the most prominent oblique fibres visible to define it but other bands were visible that showed some tightening (to a lesser extent). When found, an attachment to the lateral meniscus was identified in all specimens (Fig. 3).

## Discussion

We found a demonstrable ALL in ten out of 11 specimens and in all specimens where an intact ACL was found. This fits with previous studies where this structure has been identified both through anatomical dissection [1, 3, 10, 13, 14, 22] and magnetic resonance imaging [1, 23, 24]. Our findings were that this tissue was a part of the anterolateral capsule and that, although consisting of obvious bands orientated obliquely and parallel (and which became tight during internal rotation and varus strain), defining the anterior and posterior boundaries was, at times, fairly arbitrary. Our observation that it is a capsular thickening supports previous work [1, 3, 21, 22] and, furthermore, corresponds to our identification of attachment to the lateral meniscus that has also been reported [1, 3, 10, 21]. In contrast, Dodds et al. suggested the presence of an independent structure separate to the capsule [13]. We were unable to find any evidence to support this finding and other authors have suggested that the structure that this group identified was the capsulo-osseous layer of the ITB [1].

We were able to identify fibres, within the capsule, that became taut on internal rotation and appeared to have a role in limiting this movement. Dividing the ALL, to further investigate internal attachments and intra-articular structures, also increased anterolateral rotation. It can be argued that, having removed the ITB during dissection, the significance of this was exaggerated. However, the work of Monaco et al. showed that isolated division of this structure, without the level of dissection we performed, increased anterolateral rotation [4].

As previously mentioned, we found the dimensions of the dissected ALL were highly dependent on the technique used and it was difficult to be confident that one structure was fully separated from another. However, we used techniques that have previously been described and believe that our measurements were made in a way that is consistent with previous work. The results of other anatomical studies are summarised in Table 2.

**Table 2:** Summary of previous studies

References	Study and incidence of ALL	Femoral origin	Tibial insertion	Dimensions (mm)
Vincent et al. [21]	30 TKRs 10 cadavers 100 %	Near or on popliteus insertion (9/10 just anterior to the popliteus)	5 mm from articular margin Posterior to GT	Length: 34.1 ± 3.4 Width: 8.2 ± 1.5 Thickness: 2–3
Claes et al. [10]	41 cadavers 97 %	On lateral femoral epicondyle (anterior to LCL origin) Most superficial fibres continued over the lateral aspect of the distal femur in the direction of the lateral inter-muscular septum of the thigh The most posterior fibres blended with the proximal part of the LCL	6.5 ± 1.4 mm from articular margin 21.6 ± 4.0 mm to GT and 23.2 ± 5.7 mm anterior to FH	Length: (in neutral rotation) 41.5 ± 6.7 (at 90 flexion) 38.5 ± 6.1 (extension) Width: 8.3 ± 2.1 (mean) 6.7 ± 3.0 (at the joint line) 11.2 ± 2.5 (tibial insertion) Thickness: 1.3 ± 0.6 (at joint line)
Helito et al. [3]	20 cadavers 100 %	3.5 ± 2.1 mm distal and 2.2 ± 1.5 mm anterior to LCL origin	4.4 ± 1.1 mm from distal articular margin 38 % ± 11 % of the way from the FH to GT	Length: 37.3 ± 4.0 Width: 7.4 ± 1.7 Thickness: 2.7 ± 0.6
Dodds et al. [13]	40 cadavers 83 %	8.0 ± 5.2 mm proximal and 4.3 ± 4.9 mm posterior to the most prominent point of the lateral epicondyle Fan-like blending of fibres at the lateral aspect and from far posterior on the lateral femoral condyle	11 ± 2 mm from joint line 18 ± 3 mm from GT and 17 ± 3 mm from FH	Length: 59 ± 4 Width: 5 ± 0
Caterine et al. [1]	10 MRIs 19 cadavers 100 %	Anterodistal to LCL origin (11 specimens) Posteroproximal to LCL origin (eight specimens)	11.1 ± 2.4 mm from joint line 23.4 ± 3.4 mm from GT and 23.9 ± 5.5 mm from LCL insertion	Length: 40.3 ± 6.2 Width: 5.1 ± 1.8 (above meniscus) 8.9 ± 2.5 (below meniscus) Thickness: 1.4 ± 0.6
Stijak et al. [22]	14 cadavers 50 %	Fibres running from the lateral condyle of the femur, somewhat in front of the FCL	Midway between the GT and the FH	Length: 41 ± 3 Width: 4 ± 1 Thickness: 1

*TKR* total knee replacement, *MRI* magnetic resonance image

The tibial attachment of the ALL appears to lie just posterior to the mid-point of GT and FH. In all of our specimens (where the ALL was found) the distance to the FH from the insertion was less than the distance to GT. The average 59.5 %, we found, is supported by the results of all previous studies included in Table 2. The femoral origin has been subject to more debate, however. Caterine et al. described two variations of this origin in relation to the LCL femoral insertion [proximal-posterior (PP) or anterior-distal (AD) to the LCL] [1]. In their study, an AD origin was slightly more common; however, we found PP to be more frequent. In addition, we also found one specimen where the origin seemed to be at the same place as the LCL. However, all of these described attachments exist on a line passing through the lateral femoral epicondyle and, thus, the centre of this may be an adequate approximation.

In cases where the ALL attaches PP to the LCL, we found the ALL superficial to the LCL. This finding has been demonstrated by other groups [1, 13]. As many of the previously described extra-articular reconstructions have used a graft passing deep to the LCL, this may be one reason for over-tightening seen with these methods, and the sub-optimal results.

Our measurements are consistent with the majority of studies that suggest a length of 35–45 mm and a width less than 10 mm [1, 3, 10, 22]. However, the thickness of our measured ligament (0.87 mm) is less than previously described by Claes et al. [10] and Caterine et al. [1]. It is also significantly less than the 2–3 mm described by Vincent et al. [21]. Although similar techniques were used and the measuring apparatus appears analogous, this may represent a more thorough dissection of our specimens. Dodds et al. describe a much longer structure (with attachment further below the tibial articular surface) [13].

In summary, we were able to identify a structure that corresponds to the most frequently described ALL. Our work supports that of Caterine et al. [1] who suggest that the ALL represents a capsular thickening similar to the glenohumeral ligaments seen in the shoulder. The dimensions of our specimen mirror this group and others’ [1, 3, 10, 22]. The tibial attachment is consistently seen to lie between GT and the FH, with the femoral origin matching the description of Caterine et al. [1], and lying around the LCL attachment to the lateral femoral epicondyle. It seems logical that, given the orientation of fibres in the ALL and the tightening of this structure during internal rotation of the tibia, this structure plays a role in restraining this abnormal movement. It follows that limiting the “pivot-shift”, by reconstructing the ALL, may have a role in preventing residual instability following intra-articular ACL reconstruction. However, it should be remembered that the majority of intra-articular reconstructions have an acceptable outcome. Thus, further work is required to define the role of additional extra-articular reconstruction and the ways of determining the patients who will benefit from this. Understanding the anatomy of the ALL (and the variations that exist) is pivotal to this work. This study, therefore, is important in adding to the current literature regarding the anatomy of the ALL. The development of a consensus about the attachments of this ligament is important so that reconstructions can recreate these and provide an anatomical restraint without over-constraining the lateral compartment. Choosing to recreate the attachment to the lateral meniscus may be of benefit, and selecting a graft tissue of a similar thickness may prevent complications. Following the results of this study (and those findings that match other work), it may be that, in selected cases (and perhaps revision), reconstruction of the ALL can be found to be of additional benefit to intra-articular ACL reconstruction.
